# LncRNA2Function: a comprehensive resource for functional investigation of human lncRNAs based on RNA-seq data

**DOI:** 10.1186/1471-2164-16-S3-S2

**Published:** 2015-01-29

**Authors:** Qinghua Jiang, Rui Ma, Jixuan Wang, Xiaoliang Wu, Shuilin Jin, Jiajie Peng, Renjie Tan, Tianjiao Zhang, Yu Li, Yadong Wang

**Affiliations:** 1 School of Life Science and Technology, Harbin Institute of Technology, Harbin, Heilongjiang 150001, China; 2 School of Computer Science and Technology, Harbin Institute of Technology, Harbin, Heilongjiang 150001, China; 3 School of Software, Harbin Institute of Technology, Harbin, Heilongjiang 150001, China; 4 Department of Mathematics, Harbin Institute of Technology, Harbin, Heilongjiang, 150001, China

## Abstract

**Background:**

The GENCODE project has collected over 10,000 human long non-coding RNA (lncRNA) genes. However, the vast majority of them remain to be functionally characterized. Computational investigation of potential functions of human lncRNA genes is helpful to guide further experimental studies on lncRNAs.

**Results:**

In this study, based on expression correlation between lncRNAs and protein-coding genes across 19 human normal tissues, we used the hypergeometric test to functionally annotate a single lncRNA or a set of lncRNAs with significantly enriched functional terms among the protein-coding genes that are significantly co-expressed with the lncRNA(s). The functional terms include all nodes in the Gene Ontology (GO) and 4,380 human biological pathways collected from 12 pathway databases. We successfully mapped 9,625 human lncRNA genes to GO terms and biological pathways, and then developed the first ontology-driven user-friendly web interface named *lncRNA2Function*, which enables researchers to browse the lncRNAs associated with a specific functional term, the functional terms associated with a specific lncRNA, or to assign functional terms to a set of human lncRNA genes, such as a cluster of co-expressed lncRNAs. The *lncRNA2Function *is freely available at http://mlg.hit.edu.cn/lncrna2function.

**Conclusions:**

The LncRNA2Function is an important resource for further investigating the functions of a single human lncRNA, or functionally annotating a set of human lncRNAs of interest.

## Background

Thousands of human long non-coding RNAs (lncRNAs) have been identified and emerging studies have revealed that lncRNAs play important roles in a wide range of biological processes [1,2] and diseases [3,4]. However, functions of most human lncRNAs are still elusive. Functions of a lncRNA may be determined by loss- and gain-of-function biological experiments [5,6]. However, this is not straightforward since it is difficult to knock down a lncRNA expressed as multiple isoforms. Alternatively, computational exploration of human lncRNA functions is helpful to guide further studies on lncRNAs.

Currently, computational investigation of lncRNA functions is still at its early development stage, since it is a considerable challenge due to the characteristics of lncRNAs, e.g., many lncRNA gene sequences are not conserved and do not contain conserved sequence motifs [7], which makes it difficult to infer potential functions of lncRNAs based on their sequences alone. In addition, few available molecular interaction data of new identified lncRNAs also hamper the lncRNA functional annotations [8,9].

Since genes with similar expression patterns across multiple conditions may share similar functions [10] or be involved in related biological pathways [11], identifying protein-coding genes that are co-expressed with lncRNAs may help to assign functions to the lncRNAs. By analyzing lncRNA-mRNA co-expression pattern, Guttman et al. identified several sets of mouse lncRNAs associated with protein-coding gene sets of distinct GO functional categories [12]. In addition, two recent studies separately constructed a mouse co-expressed lncRNA-mRNA network using mouse microarray data and assigned functions to 340 and 1,625 mouse lncRNAs [13,14].

Despite accumulating insights into the mouse lncRNA functions, more than 10,000 human lncRNAs remain to be functionally characterized. Firstly, given a single human lncRNA gene, it needs to be established whether it executes crucial biological functions. Secondly, given a set of human lncRNA genes such as differential lncRNAs between cancer and normal samples, it is an important downstream task to identify significantly enriched function terms. Thirdly, given an important functional term such as a Wnt signalling pathway, how to know which lncRNAs may be involved in the pathway.

Here, based on the expression correlation between lncRNAs and protein-coding genes inferred from RNA-seq data of 19 human normal tissues, we functionally annotated 9,625 human lncRNAs with significantly enriched functional terms among the co-expressed protein-coding genes, and developed a user-friendly web interface for the lncRNA community to obtain the lncRNAs associated with a specific functional term, the functional terms associated with a specific lncRNA, or to assign functions to a set of human lncRNAs of interest.

## Methods

### Data sources

We downloaded: (1) genomic coordinates of all human lncRNA genes and protein-coding genes from the GENCODE V15 [15], (2) paired-end RNA-Seq data of 19 human normal tissues from the Human Body Map 2 project (ArrayExpress accession no. E-MTAB-513) and another study (GEO accession no. GSE30554), (3) GO assignments for the proteins of the human UniProtKB Complete Proteome from the website of the Gene Ontology Project [16], (4) 4,380 human biological pathways from the ConsensusPathDB database which integrated 12 pathway databases [17].

### Workflow of LncRNA2Function

**Figure 1:**
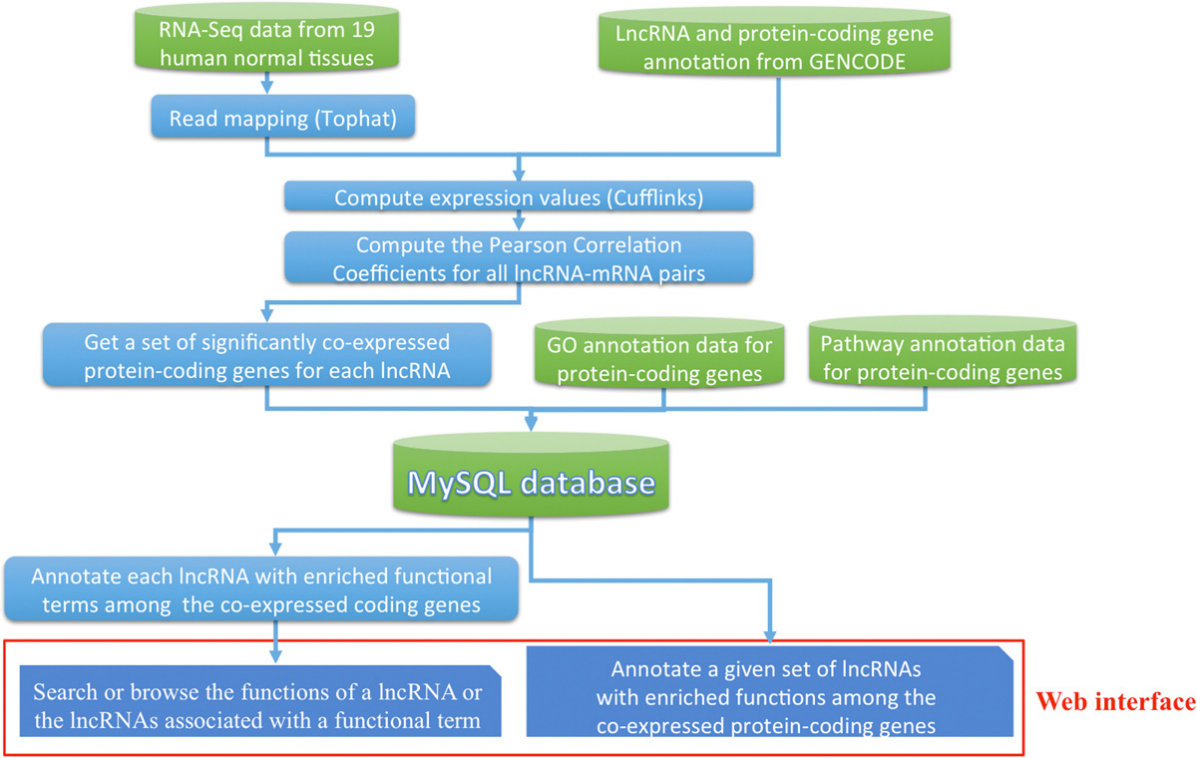
**Schematic workflow of the LncRNA2Function**.

The schematic workflow of lncRNA2Function is shown in Figure 1. Firstly, RNA-Seq reads sequenced in 19 human normal tissues were firstly mapped to human genome (hg19) using tophat with the default parameters [18], and expression values of all human lncRNA and protein-coding genes in the 19 tissues were computed using cufflinks with the default parameter [19]. Secondly, the Pearson Correlation Coefficients (PCC) of all lncRNA-mRNA gene pairs were computed, and a set of significantly co-expressed protein-coding genes was thus obtained for each human lncRNA (significant: the absolute value of the Pearson correlation coefficient >0.9 and adjusted *P-value *< 0.05). Thirdly, each lncRNA was functionally annotated with significantly enriched GO terms and biological pathways among the set of co-expressed protein-coding genes. Finally, a web interface was developed to facilitate researchers to browse or search the functions associated with a given lncRNA or lncRNAs associated with a specific function, or to functionally annotate a set of lncRNA genes of interest.

### GO and pathway enrichment analysis of human lncRNAs

Given a single human lncRNA gene, we obtained a set of protein-coding genes that were significantly co-expressed with the lncRNA. The lncRNA was then functionally annotated with significantly enriched GO and pathway terms among the set of co-expressed protein-coding genes. The enrichment analysis was separately executed for each term (denoted as T), and a *P-value *of each term was calculated by the hypergeometric test:

(1) $$p=\frac{\overset{\text{min}\left(n,M\right)}{\underset{i=m}{\sum }}\left(\begin{array}{c} M\\ i \end{array}\right)\left(\begin{array}{c} N-M\\ n-i \end{array}\right)}{\left(\begin{array}{c} N\\ n \end{array}\right)}$$

Herein, *N *is the number of all protein-coding genes in human genome, *M *is the number of protein-coding genes that were annotated in the functional term T, *n *is the number of protein-coding genes that were significantly co-expressed with the lncRNA, and *m *is the number of protein-coding genes that were both significantly co-expressed with the lncRNA and annotated in the functional term T.

For each GO term, protein-coding genes directly belong to it as well as those belong to any of its offspring terms are all considered as its annotated genes. Since the statistical analysis is not appropriate to problems with small sample size, those GO and pathway terms with less than 5 annotated protein-coding genes and those lncRNAs with less than 5 co-expressed protein-coding genes were excluded form the enrichment analysis.

Given a set of human lncRNA genes of interest, LncRNA2Function first identify a set of protein-coding genes, each of which are significantly co-expressed with one or more of the given lncRNAs across 19 human normal tissues. Then, the set of lncRNAs are functionally annotated with the enriched GO and pathway terms among the set of co-expressed protein-coding genes. If researchers input a large number of lncRNAs, the LncRNA2Function may obtain thousands of co-expressed protein-coding genes, some of which are co-expressed with only one of the lncRNAs. To improve the accuracy of functional assignments to the set of lncRNAs, users can select the protein-coding genes that are co-expressed with at least *K *lncRNAs (the *K *can be assigned based on the size of the set of lncRNAs. The default *K *is 1).

There are two commonly used methods for controlling false discovery rate (FDR), the Benjamini-Yekutieli (BY) method [20] and the Benjamini-Hochberg (BH) method [21]. The former is suitable for positively related multiple hypothesis tests whereas the later is suitable for independent multiple hypothesis tests [22]. Since the hierarchical GO terms are often dependent, we chose the BY method to correct the *P-values *from the GO enrichment analysis, and the BH method to correct the *P-values *from the pathway enrichment analysis. The significant cut-off of corrected *P-value *was set as 0.05.

## Results and discussion

### Functional annotations of a single human lncRNA

We obtained 5,232,299 significantly co-expressed pairs between 9,625 human lncRNA genes and 10,919 protein-coding genes. Each of the 9,625 lncRNAs was functionally annotated with significantly enriched GO terms and biological pathways among its co-expressed protein-coding genes. Consequently, we obtained 614,174 associations between 5,735 lncRNA genes and 3,890 GO terms, and 240,050 associations between 6,062 lncRNAs and 3,034 biological pathways. To understand the major functions of lncRNAs, we ranked GO biological processes and biological pathways according to the number of lncRNAs associated with each of them. Among the top ranked 200 GO biological processes and pathways, we found that lncRNAs play roles in many important biological processes, including defense response to bacterium, DNA packaging, meiosis, developmental process, metabolic process, cell cycle process, cell adhesion, cell differentiation, Jak-STAT signaling pathway and PI3K-Akt signaling pathway. A part of the enriched functions of lncRNAs have been validated by published studies [23-26].

### Case studies

Due to the lack of a large gold standard dataset of known human lncRNA functions, five well-studied lncRNAs were used as the examples to show the usefulness of LncRNA2Function.

### Case study 1: HOTAIR

The HOTAIR is a well-studied lncRNA. Rinn et al. found that the HOTAIR interacts with the Polycomb repressive complex 2 (PRC2) to modify chromatin and repress transcription of the HOX genes, which regulate development [27]. Niinuma et al. revealed that overexpression of HOTAIR was strongly associated with high-risk grade and metastasis among gastrointestinal stromal tumors (GIST) specimens, and knockdown of HOTAIR suppressed GIST cell invasiveness [28]. In addition, Gupta et al. demonstrated that the lncRNA HOTAIR is increased in expression in primary breast tumors and metastases, and enforced expression of HOTAIR in epithelial cancer cells leaded to altered histone H3 lysine 27 methylation, gene expression, and increased cancer invasiveness and metastasis in a manner dependent on PRC2. Conversely, loss of HOTAIR can inhibit breast cancer invasiveness [26].

To examine whether our LncRNA2Function can functionally annotate the lncRNA HOTAIR with development and metastasis-related functional terms, we applied the LncRNA2Function to the HOTAIR, and found that it was annotated with 99 GO biological processes and 33 pathways (The significant Corrected P-value cutoff is 0.05). Of the 99 GO biological processes, 77.8% (77/99) are involved in the development and morphogenesis as expected (The top 20 GO development-related biological processes are shown in Table 1), and 9.1% (9/99) are involved in the cell invasion and metastasis, such as cell migration (GO:0016477), cell adhesion (GO:0007155), biological adhesion (GO:0022610) and cell motility (GO:0048870). In addition, Of the 33 biological pathways, 72.7% (24/33) are involved in the cell invasion and metastasis (Table 2), such as focal adhesion, beta1 integrin cell surface interactions, NCAM1 interactions, Syndecan-1-mediated signaling events, PI3K-Akt signaling pathway and cell surface interactions at the vascular wall. Taken together, these results demonstrated that our LncRNA2Function can successfully recall the known functions of a well-studied lncRNA HOTAIR and suggested that it is applicable to infer potential functions of new identified lncRNAs.

**Table 1:** The top 20 biological processes assigned to the development-regulating HOTAIR by LncRNA2Function.

GO term	Background frequency	Sample frequency	P-value	Corrected P-value
System development	3253/20447	38/74	1.63E-12	2.37E-08
Anatomical structure morphogenesis	1884/20447	28/74	2.45E-11	7.10E-08
Tissue development	1183/20447	23/74	1.33E-11	7.10E-08
Embryonic skeletal system development	120/20447	10/74	1.73E-11	7.10E-08
Anatomical structure development	3717/20447	39/74	2.07E-11	7.10E-08
Skeletal system development	388/20447	14/74	1.01E-10	2.44E-07
Organ morphogenesis	790/20447	18/74	2.89E-10	4.73E-07
Multicellular organismal development	3830/20447	38/74	2.56E-10	4.73E-07
Developmental process	4248/20447	40/74	2.93E-10	4.73E-07
Organ development	2271/20447	29/74	3.63E-10	5.27E-07
Skeletal system morphogenesis	189/20447	10/74	1.54E-09	1.72E-06
Multicellular organismal process	5336/20447	44/74	1.39E-09	1.72E-06
Single-multicellular organism process	5125/20447	43/74	1.51E-09	1.72E-06
Extracellular matrix organization	204/20447	10/74	3.23E-09	3.28E-06
Extracellular structure organization	205/20447	10/74	3.39E-09	3.28E-06
Head development	52/20447	6/74	3.26E-08	2.96E-05
Embryonic skeletal system morphogenesis	91/20447	7/74	3.86E-08	3.30E-05
Single-organism developmental process	3161/20447	31/74	4.39E-08	3.54E-05
Chordate embryonic development	557/20447	13/74	9.05E-08	6.57E-05
Embryo development ending in birth or egg hatching	564/20447	13/74	1.05E-07	7.23E-05

**Table 2:** The metastasis-associated HOTAIR was annotated with metastasis-related GO and pathway terms by LncRNA2Function.

Database	Functional term	Background frequency	Sample frequency	P-value	Corrected P-value
GO	Locomotion	1022/20447	14/74	1.53E-05	0.003417
GO	Cell migration	603/20447	10/74	6.07E-05	0.010887
GO	Cell adhesion	790/20447	11/74	1.21E-04	0.020655
GO	Biological adhesion	792/20447	11/74	1.24E-04	0.020663
GO	Cell motility	664/20447	10/74	1.34E-04	0.021695
GO	Positive regulation of cell-cell adhesion	33/20447	3/74	2.30E-04	0.032376
PID	Beta1 integrin cell surface interactions	75/20447	7/74	9.92E-09	2.22E-06
Reactome	Extracellular matrix organization	102/20447	7/74	8.54E-08	9.57E-06
KEGG	ECM-receptor interaction	110/20447	7/74	1.44E-07	1.07E-05
INOH	Integrin	141/20447	7/74	7.82E-07	4.01E-05
Wikipathways	Focal Adhesion	203/20447	7/74	8.79E-06	2.46E-04
KEGG	Focal adhesion	219/20447	7/74	1.44E-05	3.58E-04
PID	Beta3 integrin cell surface interactions	47/20447	4/74	2.51E-05	5.61E-04
PID	Syndecan-1-mediated signaling events	50/20447	4/74	3.21E-05	6.53E-04
PID	Integrin cell surface interactions	58/20447	4/74	5.78E-05	0.001079
PID	Integrins in angiogenesis	73/20447	4/74	1.42E-04	0.002454
Reactome	Integrin cell surface interactions	88/20447	4/74	2.93E-04	0.004373
KEGG	PI3K-Akt signaling pathway	361/20447	7/74	3.29E-04	0.004606
Reactome	Cell surface interactions at the vascular wall	104/20447	4/74	5.53E-04	0.006566
Reactome	Signaling by PDGF	187/20447	5/74	5.86E-04	0.006566
Reactome	NCAM1 interactions	45/20447	3/74	5.79E-04	0.006566
Reactome	NCAM signaling for neurite out-growth	72/20447	3/74	0.002268	0.023101
Reactome	Platelet Adhesion to exposed collagen	22/20447	2/74	0.002848	0.025533
PID	VEGFR3 signaling in lymphatic endothelium	25/20447	2/74	0.003673	0.031645
Reactome	Basigin interactions	26/20447	2/74	0.003969	0.032935
KEGG	TGF-beta signaling pathway	92/20447	3/74	0.004537	0.036298
PID	Wnt signaling network	29/20447	2/74	0.004924	0.038039
Reactome	Degradation of the extracellular matrix	32/20447	2/74	0.005974	0.043169
Reactome	Activation of Matrix Metalloproteinases	32/20447	2/74	0.005974	0.043169
PID	Alpha4 beta1 integrin signaling events	34/20447	2/74	0.006725	0.046835

### Case study 2: HCP5

The lncRNA HCP5 was found to be associated with AIDS [29-31]. Rodriguez-Novoa et al. analyzed a total of 245 HIV patients and found a good correlation between HLA-B*5701 and HCP5 (negative and positive predictive values of 100% and 93%, respectively). Colombo et al. analyzed that 1,103 singles infected with human immunodeficiency virus (HIV) and concluded that HCP5 genotyping could serve as a simple screening tool for ABC-HSR, particularly in settings where sequence-based HLA typing is not available.

To assess whether the HCP5 can be correctly predicted to have immune-related functions, we applied our LncRNA2Function to it and found that HCP5 was annotated with 549 GO biological processes terms and 270 biological pathways. As expected, most of them are indeed immune system and response functional terms, which are strongly associated with the development of AIDS. The top 20 GO biological terms assigned to the HCP5 are shown in Table 3 while the top 20 biological pathways assigned to the HCP5 are shown in Table 4.

**Table 3:** The top 20 biological processes assigned to the AIDS-related lncRNA HCP5 by LncRNA2Function.

GO term	Background frequency	Sample frequency	P-value	Corrected P-value
Immune system process	1581/20447	208/458	1.0E-109	3.49E-105
Immune response	867/20447	148/458	1.57E-90	2.70E-86
Defense response	968/20447	144/458	2.37E-79	2.72E-75
Regulation of immune system process	879/20447	131/458	1.16E-71	9.95E-68
Regulation of immune response	527/20447	105/458	9.62E-70	6.62E-66
Response to stimulus	6195/20447	312/458	9.35E-64	5.36E-60
Cell activation	557/20447	89/458	3.11E-50	1.53E-46
Leukocyte activation	344/20447	73/458	5.00E-50	2.15E-46
Regulation of response to stimulus	2379/20447	173/458	1.23E-48	4.72E-45
Positive regulation of immune system process	522/20447	84/458	1.39E-47	4.77E-44
Response to stress	2747/20447	181/458	4.76E-45	1.26E-41
Signal transduction	3612/20447	205/458	4.25E-42	1.04E-38
Positive regulation of immune response	331/20447	63/458	4.17E-40	9.56E-37
Cellular response to stimulus	4596/20447	231/458	5.11E-40	1.10E-36
Lymphocyte activation	276/20447	58/458	1.81E-39	3.66E-36
Innate immune response	474/20447	72/458	6.13E-39	1.17E-35
Positive regulation of response to stimulus	1154/20447	106/458	6.43E-37	1.16E-33
T cell activation	176/20447	46/458	5.40E-36	9.28E-33
Single organism signaling	4081/20447	208/458	1.23E-35	1.92E-32
Immune response-regulating signaling pathway	218/20447	49/458	6.76E-35	1.01E-31

**Table 4:** The top 20 pathways assigned to AIDS-related lncRNA HCP5 by our LncRNA2Function.

Pahtway database	Pahtway name	Background frequency	Sample frequency	P-value	Corrected P-value
Reactome	Immune System	1177/20447	98/458	1.66E-30	2.14E-27
KEGG	Natural killer cell mediated cytotoxicity	219/20447	38/458	6.00E-23	3.87E-20
PID	Immunoregulatory interactions between a Lymphoid and a non-Lymphoid cell	104/20447	28/458	1.06E-22	4.54E-20
Reactome	Immunoregulatory interactions between a Lymphoid and a non-Lymphoid cell	202/20447	35/458	3.36E-21	1.08E-18
Reactome	Adaptive Immune System	772/20447	66/458	4.68E-21	1.21E-18
NetPath	TCR	252/20447	36/458	6.84E-19	1.47E-16
KEGG	Chemokine signaling pathway	195/20447	30/458	7.56E-17	1.39E-14
PID	Generation of second messenger molecules	15/20447	11/458	7.96E-16	1.28E-13
KEGG	Osteoclast differentiation	174/20447	27/458	2.20E-15	3.16E-13
PID	TCR signaling in naive CD4+ T cells	80/20447	19/458	1.03E-14	1.32E-12
PID	TCR signaling in naive CD8+ T cells	63/20447	17/458	2.63E-14	3.09E-12
PID	IL12-mediated signaling events	81/20447	18/458	1.80E-13	1.94E-11
KEGG	Cytokine-cytokine receptor interaction	291/20447	31/458	6.97E-13	6.42E-11
Reactome	Innate Immune System	542/20447	43/458	6.96E-13	6.42E-11
KEGG	Hematopoietic cell lineage	114/20447	20/458	9.26E-13	7.97E-11
KEGG	T cell receptor signaling pathway	116/20447	20/458	1.30E-12	1.05E-10
Reactome	Cell surface interactions at the vascular wall	104/20447	19/458	1.62E-12	1.23E-10
PID	Cell surface interactions at the vascular wall	42/20447	13/458	4.26E-12	3.06E-10
Reactome	Class A/1 (Rhodopsin-like receptors)	319/20447	31/458	7.85E-12	5.33E-10
PID	Fc-epsilon receptor I signaling in mast cells	64/20447	15/458	8.34E-12	5.38E-10

### Case study 3: HULC

The lncRNA HULC is highly upregulated in liver cancer and plays an important role in tumorigenesis [32]. Depletion of HULC resulted in a significant deregulation of several genes involved in liver cancer [33], and colorectal carcinomas that metastasize to the livers but not to lymph nodes experience an up-regulation of HULC in all the samples tested (n = 8), with a strong-to-moderate expression in six out of eight [34].

**Figure 2:**
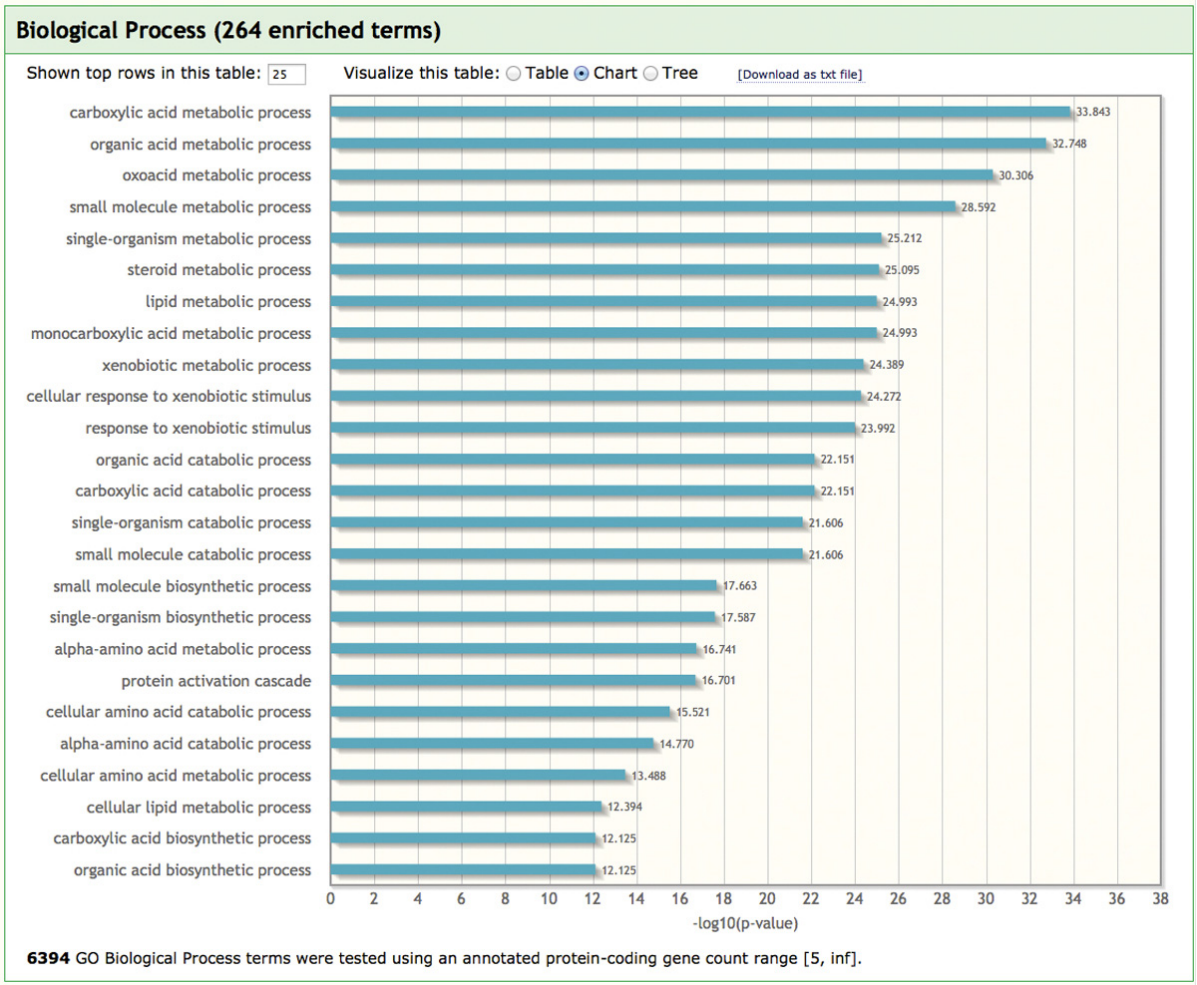
**The top 25 statistically significant enriched GO biological processes assigned to liver-related HULC are associated with the metabolic function of liver**.

To examine whether the HULC was predicted to have liver-related functions, we analyzed it using our lncRNA2Function. Expectedly, LncRNA2Function also works well to functionally annotate the HULC. The results showed that it was annotated with 373 GO biological processes and 383 biological pathways (the significant P-value cutoff is 0.05). Of the 373 GO biological processes and 383 pathways, over 80% are involved in the known liver-related biological functions, such as metabolic function, bile secretion, lipid transport and homeostasis, cholesterol homeostasis, regulation of blood coagulation, protein-lipid complex subunit organization, detoxification, Immune defense and complement activation. The Figure 2 shows the top 25 GO functional terms assigned to the HULC, and the Table 5 shows the top 20 pathways enriched in protein-coding genes that are co-expressed with the liver-related lncRNA HULC.

**Table 5:** Top 20 pathways enriched in protein-coding genes that are co-expressed with the liver-related lncRNA HULC.

Pahtway database	Pahtway name	Background frequency	Sample frequency	P-value	Corrected P-value
Reactome	Metabolism	1394/20447	128/390	6.17E-54	6.23E-51
KEGG	Metabolic pathways	1256/20447	109/390	7.95E-43	4.01E-40
KEGG	Retinol metabolism	69/20447	29/390	5.66E-32	1.91E-29
KEGG	Complement and coagulation cascades	87/20447	30/390	6.18E-30	1.56E-27
EHMN	Androgen and estrogen biosynthesis and metabolism	90/20447	30/390	2.02E-29	4.07E-27
Reactome	Biological oxidations	151/20447	36/390	2.71E-29	4.55E-27
KEGG	Drug metabolism - cytochrome P450	76/20447	28/390	5.84E-29	8.41E-27
KEGG	Metabolism of xenobiotics by cytochrome P450	87/20447	29/390	1.78E-28	2.24E-26
KEGG	Chemical carcinogenesis	86/20447	28/390	3.33E-27	3.74E-25
EHMN	Tyrosine metabolism	113/20447	30/390	4.29E-26	4.33E-24
EHMN	Xenobiotics metabolism	54/20447	23/390	9.33E-26	8.56E-24
Reactome	Metabolism of amino acids and derivatives	159/20447	32/390	1.09E-23	9.20E-22
Wikipathways	metapathway biotransformation	190/20447	34/390	2.43E-23	1.88E-21
EHMN	Linoleate metabolism	75/20447	24/390	2.81E-23	2.03E-21
Wikipathways	cytochrome P450	68/20447	23/390	5.40E-23	3.63E-21
Wikipathways	Complement and Coagulation Cascades	60/20447	22/390	5.97E-23	3.77E-21
EHMN	Leukotriene metabolism	102/20447	26/390	2.87E-22	1.70E-20
Reactome	Phase 1 - Functionalization of compounds	74/20447	23/390	5.01E-22	2.81E-20
KEGG	Drug metabolism - other enzymes	53/20447	20/390	2.85E-21	1.51E-19
Reactome	Xenobiotics	15/20447	13/390	3.68E-21	1.86E-19

### Case study 4: H19

H19 is an important lncRNA that play roles in the infertility [35] and multiple cancers such as breast cancer [36,37], cervical cancer [38], liver cancer [39,40] and bladder cancer [41]. For example, Korucuoglu et al. revealed that H19 expression was lower in the infertility group as compared to the control group (4-fold change, P < 0.0001), and Lottin et al. showed that over-expression of H19 transcript is associated with cells exhibiting higher tumorigenic phenotypes and promotes tumor progression.

We applied the LncRNA2Function to the lncRNA H19 and found that it was annotated with 6 GO biological processes and 31 biological pathways. The GO terms includes female pregnancy (GO: 0007565), estrogen biosynthetic process (GO:0006703), growth hormone receptor signaling pathway (GO:0060396), cellular response to growth hormone stimulus (GO:0071378) and JAK-STAT cascade involved in growth hormone signaling pathway (GO:0060397), which suggest that H19 may play roles in infertility or breast cancer by participating in these biological processes. In addition, the cancer-related lncRNA H19 was correctly annotated with many important caner pathways, such as PI3K-Akt signaling pathway, GPCR signaling-G alpha s Epac and ERK pathway, Nuclear signaling by ERBB4 pathway, Akt signaling pathway and JAK-STAT-Core cancer pathway. These results suggest that our LncRNA2Function correctly recall the known functions of H19.

### Case study 5: PCA3

The lncRNA prostate cancer antigen 3 (PCA3) is a highly specific biomarker upregulated and plays crucial roles in prostate cancer (PCa) [42-45]. Clarke et al. found that up-regulation of two new PCA3 isoforms in PCa tissues improves discrimination between PCa and benign prostatic hyperplasia (BPH). In 2012, the US Food and Drug Administration approved the use of the lncRNA PCA3 for the detection of prostate cancer.

To test whether our LncRNA2Function can annotate the PCA3 with prostate-related functions, we applied the LncRNA2Function to the PCA3. LncRNA2Function first identified 77 protein-coding genes that are co-expressed with the PCA3 and then annotated it with only one pathway named 'Regulation of Androgen receptor activity' (corrected P-value: 0.020385). This pathway has 62 genes, which includes 4 protein-coding genes that are co-expressed with the PCA3. These four genes are HOXB13, KLK3, KLK2 and SPDEF that have been validated to be useful in the diagnosis and monitoring of prostatic carcinoma and be suitable target for developing specific cancer therapies. Consequently, lncRNA2Function can correctly predict the functions of PCA3 by its co-expressed protein-coding genes.

### Functional annotation for a set of human lncRNAs

High-throughput genomic technologies like lncRNA microarray and RNA-Seq usually generate hundreds of candidate lncRNA genes of interest, such as a cluster of co-expressed lncRNA genes across multiple conditions or a set of differentially expressed lncRNAs between cancer and normal samples. To manually map each lncRNA to functional terms is by far a simple task. Therefore, how to identify significantly enriched functions among the set of lncRNAs is an important downstream task for interpreting high-throughput experimental data.

As a proof-of-concept, a set of liver-specific lncRNAs and a set of heart-specific lncRNAs inferred from RNA-Seq data of 19 human normal tissues were used as examples to show the functionality of our lncRNA2Function system in annotating a set of lncRNAs of interest, respectively. As expected, lncRNA2Function correctly assigned the functional terms to the two distinct sets of lncRNAs. Users can test these two sets or their own lncRNA sets at our 'LncRNA set analyzer' web interface http://mlg.hit.edu.cn/lncrna2function/lncrna_enrich.jsp.

### Web interface of LncRNA2Function

**Figure 3:**
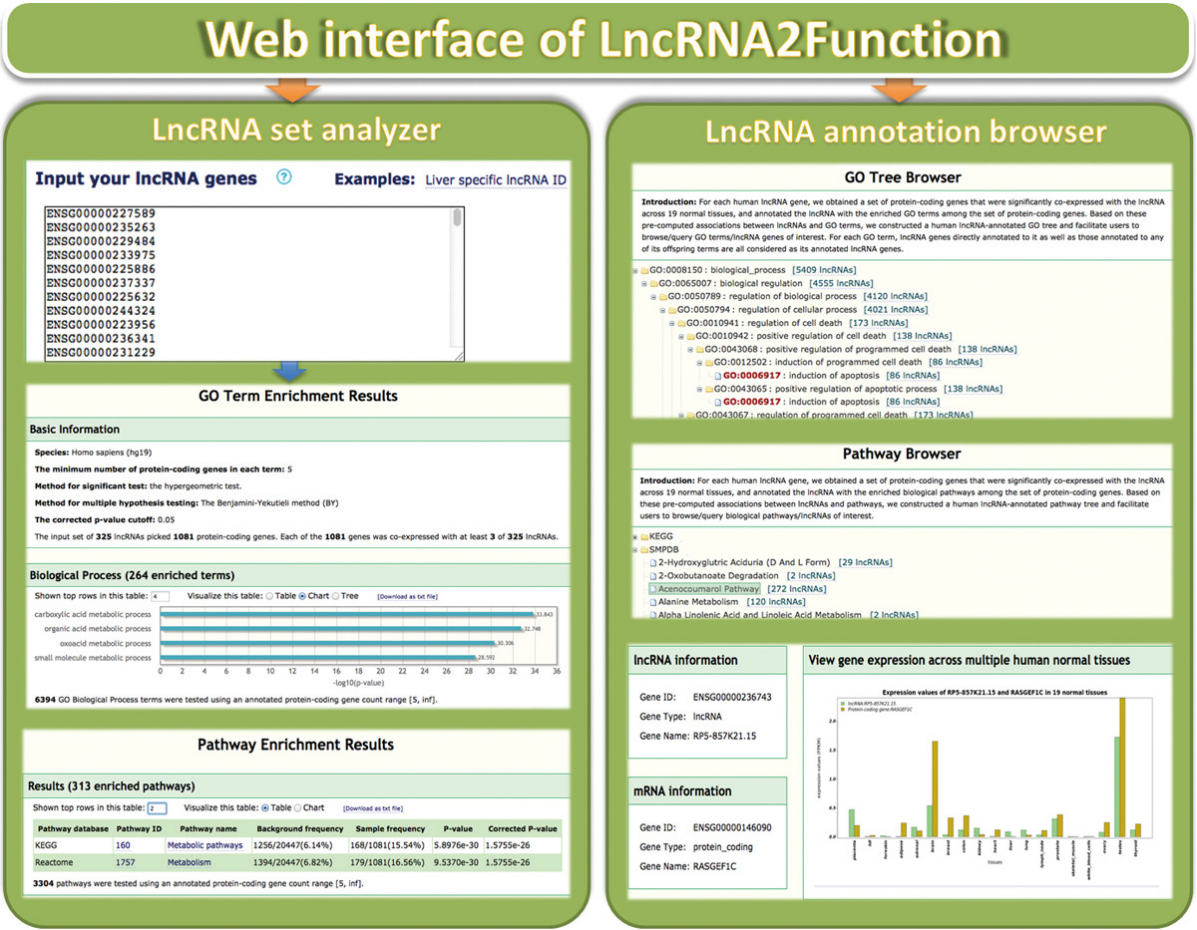
**Screenshot of web interface of LncRNA2Function**.

To facilitate researchers to access the functional annotations of lncRNA genes, we developed a web interface named 'LncRNA annotation browser', which is a user-friendly interface to browse or search lncRNAs associated with a specific functional term, or functional terms associated with a given lncRNA. To enable researchers to analyze a set of lncRNA genes of their interest, we implemented a web interface titled 'LncRNA set analyzer', which can help investigators to annotate a set of lncRNAs with Gene Ontology and 4,380 biological pathways curated from 12 pathway databases. In addition, we developed a web interface titled 'LncRNA expression viewer' to facilitate investigators to graphically view the expression dynamics of genes across multiple human normal tissues. Users can not only view expression value of a single lncRNA or protein-coding gene across 19 human normal tissues, but also simultaneously view the expression index of both lncRNA and protein-coding genes to learn about whether they are co-expressed across the 19 tissues. Furthermore, we provide a submission page that allows other researchers to submit known functional annotations of lncRNAs that are not documented in our LncRNA2Function system (Figure 3). They do not have to be an author on the original study to submit a record. Once approved by the submission review committee, the submitted records will be made available to the public in the coming release. LncRNA2Function is freely accessible at http://mlg.hit.edu.cn/lncrna2function.

## Conclusions

Thousands of human lncRNAs have been identified in recent several years, while the vast majority of the lncRNAs remain to be functionally characterized. In this study, we functionally annotate 9,625 human lncRNAs with the enriched functions among the protein-coding genes that are co-expressed with each lncRNA. Furthermore, we developed a web interface, which facilitates researchers to search the functions of a specific lncRNA or the lncRNAs associated with a given functional term, or annotate functionally a set of human lncRNAs of interest. The *lncRNA2Function *will become an important tool for investigating functions of human lncRNAs.

## Competing interests

The authors declare that they have no competing interests

## Authors' contributions

YW and YL conceived and designed the experiments. QJ, RM, XW, SJ, TZ, RT and JP performed the experiments and analyzed the data. QJ and JW designed and developed the web interface. QJ, YW and YL wrote the paper.
